# A systematic review of patient reported factors associated with uptake and completion of cardiovascular lifestyle behaviour change

**DOI:** 10.1186/1471-2261-12-120

**Published:** 2012-12-08

**Authors:** Jenni Murray, Cheryl Leanne Craigs, Kate Mary Hill, Stephanie Honey, Allan House

**Affiliations:** 1 Academic Unit of Psychiatry and Behavioural Sciences, Leeds Institute of Health Sciences, The University of Leeds, Charles Thackrah Building, 101 Clarendon Road, Leeds, LS2 9LJ, UK

**Keywords:** Health behavior, Primary health care, Cardiovascular diseases, Primary prevention, Lifestyle, Secondary prevention

## Abstract

**Background:**

Healthy lifestyles are an important facet of cardiovascular risk management. Unfortunately many individuals fail to engage with lifestyle change programmes. There are many factors that patients report as influencing their decisions about initiating lifestyle change. This is challenging for health care professionals who may lack the skills and time to address a broad range of barriers to lifestyle behaviour. Guidance on which factors to focus on during lifestyle consultations may assist healthcare professionals to hone their skills and knowledge leading to more productive patient interactions with ultimately better uptake of lifestyle behaviour change support. The aim of our study was to clarify which influences reported by patients predict uptake and completion of formal lifestyle change programmes.

**Methods:**

A systematic narrative review of quantitative observational studies reporting factors (influences) associated with uptake and completion of lifestyle behaviour change programmes. Quantitative observational studies involving patients at high risk of cardiovascular events were identified through electronic searching and screened against pre-defined selection criteria. Factors were extracted and organised into an existing qualitative framework.

**Results:**

374 factors were extracted from 32 studies. Factors most consistently associated with uptake of lifestyle change related to support from family and friends, transport and other costs, and beliefs about the causes of illness and lifestyle change. Depression and anxiety also appear to influence uptake as well as completion. Many factors show inconsistent patterns with respect to uptake and completion of lifestyle change programmes.

**Conclusion:**

There are a small number of factors that consistently appear to influence uptake and completion of cardiovascular lifestyle behaviour change. These factors could be considered during patient consultations to promote a tailored approach to decision making about the most suitable type and level lifestyle behaviour change support.

## Background

Many developed countries are witnessing increasing rates of diabetes and cardiovascular diseases. Evidence to support adoption of healthy lifestyles in the prevention and management of these and other long-term conditions is strong [1-3]. As an approach to managing cardiovascular disease risk, promotion of healthy lifestyles is arguably the preferred first line option over medication, which although effective in reducing cardiovascular risk [4], frequently has side-effects [5] and offers benefits only with continued adherence. A number of countries, including the UK, [6] now offer cardiovascular health checks through systematic screening programmes based in primary care. A variety of modalities exist but commonly they offer individuals a chance to modify their lifestyle behaviours and so invest in their future health.

Despite its importance, many individuals fail to engage in activities designed to help them to achieve a healthy lifestyle. For example, only around one third of patients experiencing a cardiac event take up cardiac rehabilitation [7] and up to one quarter of participants in commercial weight management programmes drop out [8]. The individual factors that can dissuade individuals from achieving lifestyle change are multiple and inter-related but broadly cluster as social, psychological and practical barriers. Compounded by the physically addictive nature of some behaviours, lifestyle change is challenging, requiring support and personal determination. The potential myriad of personal barriers to lifestyle change presented to health care providers during consultations are challenging to address [9]. Lack of skills and knowledge combined with short consultation times [10,11] have the potential to generate generic responses that fail to meet individual needs. So, how can health care services develop a more skilled and focused approach to lifestyle behaviour change for individuals at high risk of cardiovascular events?

One option is to systematically target individually perceived key barriers to healthy lifestyles. Interventions specifically aimed at increasing initiation of lifestyle change [12-14] or uptake of associated programmes [15,16], tend to address patient perceived barriers as they arise in consultation and the concept of barrier *removal* has been applied naively as a component of a more complex approach. None have specifically developed a barriers-based framework as the core of the intervention. An alternative approach would be to use knowledge of barriers and facilitators to tailor care pathways such that existing resources were more closely matched to individual need. Such an approach requires greater clarity on key influences on lifestyle behaviour change.

As the first step in developing such an approach we conducted a review of the qualitative literature reporting patient experiences of lifestyle change [17]. This revealed 348 factors that patients considered to influence lifestyle change. These were aggregated into 20 categories in which we identified five key themes: emotions; psychological beliefs; information / communication needs; support from family and friends; and transport and other costs) [17]. While this information is clinically valuable, its routine integration into relevant patient consultations requires more robust evidence. As the next step in developing a ‘lifestyle referral assessment’ we considered it necessary to identify which of the many reported influences were not just perceived by patients to be important, but have been shown to be associated with lifestyle behaviour change. We hypothesised that key influences identified in the earlier qualitative review would also likely predict uptake of lifestyle behaviour change. Previous published literature reviews on predictors of lifestyle behaviour change have focused on uptake of cardiac rehabilitation [18,19]. Through a review of quantitative observational correlation studies, we aimed to clarify more explicitly which patient centred-factors act on uptake *and* completion of formal lifestyle change programmes, attempting to draw on broader literature in for example diabetes and hypertension. Our review fulfils a dual need, to provide guidance to health care staff when referring individuals to lifestyle support (increasing uptake), as well as those delivering formal programmes (maximising retention).

## Methods

### Searching and study selection

An electronic search strategy was developed by the authors and run in Medline, HMIC, OVID Nursing, PsycInfo and Embase from 1980 to February 2010 (available from authors on request). Search terms included cardiovascular diseases, attitude to health and prevention. The start date of 1980 was selected to reflect early influential public health policies such as the 1984 BMA drivers to commence NHS smoking cessation programmes (which started in 1998) alongside existing and expanding telephone help lines. Search results were imported into Endnote (Version X2) and independent reviewers (JM, CC, SH, KH) undertook title and abstract screening against predefined selection criteria. Reference lists of eligible studies were hand-searched for additional systematic reviews. Stages of the review process involving multiple reviewers (JM, CC, SH, KH) included group discussions to resolve any discrepancies.

The inclusion criteria were primary or secondary quantitative research studies examining uptake (attending at least one session) or completion (attending all sessions) of lifestyle behaviour change programmes in adults (>18 years of age) having experienced angina, myocardial infarction or transient ischemic attack, or with hypertension, diabetes type II, coronary artery disease or hypercholesterolemia. Studies were excluded if they: investigated compliance with medication for cardiovascular risk management or long term maintenance of lifestyle change (these are arguably separate bodies of research and their inclusion would result in an unwieldy report); focused on a selected population such as mental health patients (regarded as a group with specialist needs); were culturally unrepresentative of the main ethnic groups residing in Europe; included only stroke, chronic obstructive pulmonary disease, peripheral artery disease, and heart failure patients (on the basis that physical limitations would have major influences on lifestyle choices) or; consisted entirely of Diabetes type I patients (most likely to involve adolescents). Primary research studies were further required to report statistical effects (as p values or 95% confidence intervals) of investigated factors on uptake or completion of lifestyle behaviour change. Likewise, reviews were included if they were statistical meta-analyses of selected factors or narrative reviews with clear reporting of statistical effects of factors from included identifiable primary research studies. If necessary, authors were contacted to clarify reported data.

### Quality assessment

A quality assessment on primary research studies was conducted using a 14-item adapted version of the STROBE guidelines for assessing observational studies [20]. One reviewer assessed all papers, with a second reviewer assessing a subset of papers. Studies were deemed poor in quality and were rejected from the review if they met less than 50% of the quality criteria. Studies meeting between 50% and 70% were regarded as adequate and those scoring more than 70% were good.

Quality assessment of identified reviews comprised four criteria that were common to two existing meta-reviews [21,22] and a previously published checklist [23]. Reviews lacking adequate methodological description to allow us to apply these criteria were rejected.

### Data extraction

Data extraction was performed independently on eligible studies by two reviewers (CC and JM). Study details were extracted and the following information related to findings was recorded: outcome type (uptake or completion), factor (e.g., age, depression, marital status), statistical method, statistical significance (p<0.05 was deemed statistically significant), direction of relationship (barrier or facilitator to lifestyle change).

Extracted factors were organised into a framework derived from our previous review [17] comprising 20 categories. We split the categories into those that were regarded as key themes (mood, information and communication, support from friends and family, psychological beliefs, and transport and other costs) and other categories. An additional category covering demographic factors was also included. Extracted organised data were checked by a second reviewer. All outcomes were recorded in Microsoft Excel 2007.

### Analysis

A narrative review was conducted due to the heterogeneity between studies, in terms of the clinical populations and diversity in outcomes and outcomes assessment. Data were organised according to whether they appeared to deter, facilitate or had no relationship with either uptake or completion of lifestyle programmes. Investigated factors for which there was no evidence of relationship with uptake or completion of lifestyle programmes were recorded separately.

Finally we compared the factors reported in the framework from the qualitative literature with those examined in the quantitative studies to see whether the patient reported factors were represented in the statistical studies.

## Results

The electronic search generated 16,802 hits (after de-duplication).

### Systematic reviews and quality assessment

Ten reviews (seven identified from electronic database searching and three through hand searching) underwent full paper screening. Six reviews were rejected as they either provided no description of their methods or reported very limited statistical results from included primary studies [18,24-28]. One further review was rejected because it failed to indicate which primary studies reported non-significant predictors of uptake at cardiac rehabilitation [18]. Therefore three systematic reviews [19,29,30] reporting predictors of uptake at cardiac rehabilitation were eligible for inclusion. Two reported meta-analyses of individual predictors of uptake (attendees versus non-attendees) of cardiac rehabilitation [29,30]. The third [19] was a narrative review summarising findings from 15 studies [31-45] on a range of variables. The individual findings from the third review were extracted and are reported alongside the findings from the identified primary studies. A quality assessment of these individual studies was not undertaken.

### Primary studies and quality assessment

In total 166 papers underwent full paper screening of which 33 [46-78] met the inclusion criteria. Reasons for rejection included: paper predating or reported in the review by Cooper et al. [19] (n=72), paper not assessing cardiovascular prevention initiatives (n=19), outcomes were medication adherence or maintenance with no mention of uptake or completion (n=20); absence of statistical analysis between groups (n=18 papers), and other (n=4). The main characteristics of the primary studies are summarised in Table 1.

**Table 1:** Main characteristics of included 32 primary studies

**Study characteristics**	**Total [references]**
**Outcome:**	
Uptake in CR^a^	24 [46,48-57,60,61,63-67,70,72][74-77]
Completed CR	11 [47,51,55,59,62,68,69,71,76,78]
**Design:**	
Cross sectional	7 [49,53,62,67,68,72,74]
Cohort	24 [46-48,50-52,54-57,59-61,63-66][69-71,73,75,76,78]
RCT	1 [77]
**Prospective/retrospective:**	
Prospective	23 [46,48-52,54-56,59-61][63-66,69,70,73,74,76-78]
Retrospective	9[47,53,57,62,67,68,71,72,75]
**Country**:	
USA	5 [47,55,59,68,75]
UK	7 [48,63,64,70,73,74,78]
Australia/New Zealand	9 [49,51-54,60,67,76,77]
Canada	4 [46,56,57,62]
Rest of Europe (Sweden, Denmark, Poland)	4 [50,65,66,72]
Middle East	3 [61,69,71]

CR – cardiac rehabilitation; RCT – randomized controlled trial.

^a^Whitmarsh et al., 2003 [74] combined non-attendees with poor attendees in their analysis and as the majority group were non-attendees the study has been categorised as ‘uptake’.

One paper which met the inclusion criteria was found to be poor in quality (scoring 5/14 (36%) and was rejected [58]. Five papers were considered to be of adequate quality [61,62,72-74] and 27 were good (Additional file 1).

### Summary of findings

In total 374 factors were extracted from the 32 included primary studies. Twenty-three studies reported 253 factors relating to uptake, with the remaining 121 factors from 11 studies relating to completion. Five factors were co-reported in two systematic reviews and were therefore excluded. Three studies [51,55,76] reported on both uptake and completion of programmes. All studies were concerned with cardiac rehabilitation despite the broad scope of the terms defined in the electronic search strategy. Most studies reporting uptake failed to define what was meant by attendance or participation but all studies included a comparator group of non-attendees. In two studies, completion of programmes was defined as completing at least half [76] or two-thirds [55] of all the sessions with the comparator groups for these variables being those who dropped out earlier (as opposed to those who did not start the programme). All other studies reported completion as attendance at all sessions.

### Key themes

Key themes that contained factors most consistently associated with uptake at lifestyle programmes were ‘friends and family support’, ‘transport and other costs’, and ‘psychological beliefs’ (Table 2). Problems with transport, perception of greater consequences to illness and attribution of more symptoms to illness were most consistently predictive of uptake. Absence of a partner, lack of employment, transport / distance problems, low self-efficacy and perceptions of less control of the illness were commonly predictive of non-uptake. Earlier studies suggest that lower educational attainment may predict non-uptake, however this has not been supported by the majority of more recent studies. Anxiety and depression were the most frequently reported emotional factors. None of the studies suggested that anxiety deters uptake of lifestyle change with one (out of eight) even suggesting it to be facilitative. Just under half the studies examining the relationship between depression and uptake found that affected individuals may be less likely to start a lifestyle programme. One study [74] indicated a facilitative role of depression in uptake, however it was not found to be independently predictive. Programme drop-out appears to be consistently associated with the presence of depression (Table 3).

**Table 2:** Studies reporting factors (organised into key themes) relating to uptake of lifestyle programmes

**Factors**	**References of studies examining uptake**		
	**Facilitates**	**Deters**	**No association**
**Emotions**			
Increased anxiety	56**		31, 35, 38, 50^a^, 52, 60, 70
Depression		31**^b^, 38, 50, 55**, 61, 67	31 ^b^, 44, 35, 52, 60, 66, 70
Stress		50**, 54	52
Less distress, lower mental QOL, denial, greater health concerns, higher role resumption		43, 61, 72, 35, 37	
**Psychological beliefs**			
Illness less attributed to lifestyles, increased denial of severity of illness		33**, 31**	
Less control/ cure over course of illness/ lower self efficacy		30^m^, 56**	54, 37
More symptoms attributed to illness / better understanding of illness/ illness has greater consequences	30 ^m^		
**Information & communication**			
Less education		31**, 35**, 34**, 36, 41, 61	38, 50, 60, 66, 76, 77
Less awareness of blood pressure level		67	33
Less awareness / knowledge of total cholesterol level or recommended activity levels		33**, 67	
**Friends & family support**			
Not married / not living with a partner / being single		29^m^ , 34, 38, 52**^a^, 53**, 65**	35, 36, 49, 50, 52^b^, 60, 66,77
**Transport & related costs**^**†**^			
Longer commute time		31**,60**	
Greater distance from venue		49	40, 50, 60
Problems with transport, rurality		51**, 76**^c^, 36**, 45**	
Occupation type - blue collar (vs white)		31, 65	77
Unemployed / retired/home maker	65	33, 35, 34**, 38**, 50, 54**, 76	32, 36, 41, 49, 52, 67, 77
Higher income	61, 65**	35, 56**	50, 53
Having health insurance	77		

**Independently significant.

Regression analysis not reported [32,37,43,44,46,48-50,57,66,67,72,73].

^a^ anxiety trait; ^b^ Ades and colleagues [31] measured depression prior to and during hospitalisation and found conflicting results; ^c^ in men only; ^m^ meta-analysis.

† This key theme was derived from two individual categories [17].

**Table 3:** Studies reporting factors (organised into key themes) relating to completion of lifestyle programmes

**Factors**	**References of studies examining completion**		
	**Facilitates**	**Deters**	**No association**
**Emotions**			
Increased anxiety	74**	55, 73	
Depression	74	47, 55, 73, 78**^a^	
On antidepressant medication		47, 62	
Greater neuroticism		55**	
Emotion focused coping	74**		
Greater optimism	55**		
**Psychological & spiritual beliefs**			
Greater personal control & less treatment control		78**	
Illness has greater consequences / Timeline (acute/chronic) and (cyclical)	78**		
More symptoms attributed to illness		78	
**Information & communication**			
Less education		71**	69, 76, 74
**Friends & family support**			
Not married/not living with a partner/being single		47, 62**	69, 76
**Transport & related costs**^**†**^			
Unemployed / retired/home maker	69	68, 76**^b^	74

**Independently significant.

Regression analysis not reported [73].

^a^ combined measure for depression and anxiety; ^b^ in men only (deters prior to hospitalisation but not during hospitalisation).

Within the five themes there were a number of factors that appeared to have no evidence of any relationship with uptake or completion of programmes. These included for example, knowledge of smoking cessation, living alone and occupation (Table 4).

**Table 4:** Factors from key themes showing no evidence of relationship with uptake / completion of CR programmes [references]

**Category**	**Factors**
**Emotions**[33,36-38,50,52,74]	*Uptake* - Anxiety stait; alexithymia; distress caused by symptoms; emotional health (profile of mood state; post traumatic stress disorder, self motivation
	*Completion* - problem focused coping; maladaptive coping
**Psychological beliefs**[30,36,37,78]	*Uptake* – Overall health beliefs; multidimensional health locus of control; illness perceptions personal control; illness perceptions treatment control; illness perceptions timeline
	*Completion* - Emotional representations; time cyclical (symptoms change)
**Information and communication**[67]	*Uptake* - Knowledge of smoking recommendation
**Family & friends support**[49,60,64,76]	*Uptake* - Living alone; relationship difficulties
	*Completion* - Living arrangements
**Transport & cost**[47,48,60,70,74,76]	*Uptake* – occupation; transport cost and financial difficulty; distance from centre
	*Completion* – Transport problems; income; occupation, ‘practical barriers’ (broadly defined)

Concordance between the lay concepts that defined the qualitative framework [17] and the factors that were reported in the quantitative observational studies appeared good for only one of the five key themes (‘transport and other costs’). For example, emotional factors in the qualitative framework included fear, lack of motivation, confidence and embarrassment while the quantitative studies reported only on anxiety and depression. Factors relating to ‘friends and family support’ were limited to partner status in the quantitative data as compared with the qualitative framework which reported more nuanced factors relating to the quality of support.

### Other categories

Numerous factors relating to ‘physical wellbeing’ were reported in the quantitative studies. Despite this, the relationships between uptake of cardiac rehabilitation and either previous cardiac events, cardiac procedures or clinical risk factors remain unclear (Table 5). In contrast, the presence of co-morbid long-term conditions and poor physical functioning appears to deter uptake. While most of the evidence on body weight / mass index indicates that this has no relationship with uptake, there may an increased likelihood of overweight individuals dropping out of programmes (Table 6). Many of the factors relating to ‘physical wellbeing’ showed no evidence of relationship with either uptake or completion of lifestyle change programmes (Table 7). There was a paucity of studies reporting the relationship between uptake or completion of lifestyle change support and factors relating to ‘referrals’, ‘culture’, ‘social support’, ‘the role of the health care professional’, ‘attitudes to rehabilitation’, ‘attitudes to exercise’ and ‘balancing and integrating health care needs with everyday life’. Current smoking status (the only factor relating to ‘personal choices and cultural preferences’) produced conflicting results with regards to uptake and completion. As with ‘physical wellbeing’ there has been extensive research into the relationship between uptake, and both gender and age. Although there may be a trend towards non-uptake among females and older people, the majority of studies suggested no relationship.

**Table 5:** Studies reporting factors from other categories relating to uptake of cardiac rehabilitation

**Factors**	**References of studies examining uptake**		
	**Facilitates**	**Deters**	**No relation**
**Language barriers**			
Non-English speaking background / less likely to speak English		45**	52, 60, 76, 77
**Physical wellbeing**			
(History of) CHD	51	38, 41, 75	33, 66, 67, 70, 77
History of neurological / cognitive impairment		45**	
ACS (compared to IHD)	61		
Angina pain / MI	65, 51		32
Previous cardiac event or cardiac procedure^a†^	34, 41, 54, 65, 67, 75	34, 38**, 40**, 45**, 76**	31, 32, 33, 52, 65, 67, 76
Presence of clinical cardiac risk factors^b‡^	32, 34, 65^c^, 75, 76, 77	34, 67, 75	19, 35, 38, 45, 50, 52, 60, 67, 77, 76
Co-morbid long-term conditions^d^		31, 45**, 75, 67, 76^e^	35, 38, 42, 50, 52, 60, 65, 77
Family history of CHD	34, 76	75	52, 77
Increased weight & body mass index	60, 75		33, 50, 60, 67, 76, 77
Various indicators of cardiac condition^f^	75	38, 40, 65**,	50
Less frequent diagnosis of angina		41	
Poorer physical functioning/physical QOL		35**, 61**	36, 50, 60
On medication for cardiac problems	38, 40, 65	67^g^	67^h^
**Balancing and integrating health care needs with daily life**			
Family obligations		50	
**Referrals**			
Not receiving an outpatient appointment		40	
**Culture**			
Foreign citizen		65, 77	
Jewish (compared to Muslim)	61		
**Social support**			
Practical support	64**		
Less social support		36	37, 56
Medium to large social network (versus small)	64		
**Role of health care professional**			
Perceived strength of physician recommendation / involvement of a cardiologist	31, 42, 75		
**Attitudes to rehabilitation**			
CR more suited to younger and more active individuals		48	
CR is necessary/ intention to attend, previously attended CR	33, 36, 48, 51		
**Attitudes to exercise**			
Sedentary lifestyle / less regular exercise	52	38, 35, 67	32, 33, 76
**Personal choices and cultural preferences**			
Current smoking	34, 38, 45, 75	50, 67	32, 33, 35, 52, 60, 67, 77
**Demographics**			
Greater deprivation		36, 38, 40, 42	70
Female		34**, 35, 37, 38, 39**, 46, 57, 75, 77	31, 33, 36, 40, 42, 45, 49, 50, 52, 53, 56, 60, 65, 66, 67, 70
Older age	36, 77	31, 33**, 37, 38, 39**, 45**, 57, 75, 77	31, 33, 36, 40, 42, 49, 50, 52, 53, 56, 60, 65, 66, 67, 70
Age between 55–74 years (compared with younger and older groups) / being a pet owner	54		

Regression analysis not reported [46,48-50,57,66,67,72,73].

^**^ Independently significant.

^†^Cardiac procedures: Reperfusion (not otherwise specified), percutaneous coronary intervention, coronary bypass surgery, electrical cardioversion.

^‡^ Clinical cardiac risk factors: hypertension and hyperlipidemia (includes stated high cholesterol).

^a^Evenson and colleagues [34] had conflicting results for having had an event versus having had a procedure. Nielsen et al. [65], Worcester et al. [76] and Redfern et al. [67], had conflicting results for different cardiac procedures.

^b^ Evenson et al. [34] reported conflicting results for hypertension and hyperlipidemia with uptake correlated with (more likelihood of) hyperlipidemia) and non uptake correlated with (more likelihood of) hypertension.

^c^ Raised LDL cholesterol facilitating uptake in women only.

^d^ Includes diabetes, COPD, asthma, other undefined.

^e^ Men with diabetes (not observed in women).

^f^ Various indicators of cardiac condition included: ECG T-wave inversion (independently significant and tachycardia (not independently significant) [50]; NHAR classification (possible versus probable AMI) [40];Greater ejection fraction [50,75]; More severe cardiac infarction [38,46].

^g^ One (statin) of eight different medication types (e.g. anti-hypertensives) was negatively associated with attendance. All others were not associated with attendance.

**Table 6:** Factors reported in other patient centred categories relating to completion of programmes

**Factors**	**References of studies examining completion**		
	**Facilitates**	**Deters**	**No relation**
**Physical wellbeing**			
Previous cardiac event or cardiac procedure	47, 62		76
Presence of clinical cardiac risk factors^a^		68^b^, 69^b^	
Increased body weight / body mass index		62,68, 69**	55, 76
Poorer physical functioning/physical QOL		55, 68	
Various indicators of cardiac condition^b^	62, 73		55
On medication for cardiac problems		62	
**Culture**			
Ethnicity (white race)	47		68
**Attitudes to exercise**			
Less regular exercise		76** (females)	68, 69
**Personal choices and cultural preferences**			
Current smoking	47	62, 68, 69, 76	71
**Demographics**			
Greater deprivation			51
Female	68, 69	47, 62, 78**	71
Older age	47, 55, 62, 69, 73		68, 71, 76, 78
Age between 55–74 years (compared with younger and older groups)	51, 59		
Height		69	

Regression analysis not reported [73].

^†^Cardiac procedures: Reperfusion (not otherwise specified), percutaneous coronary intervention, coronary bypass surgery, electrical cardioversion.

^a^ High risk status (other clinical risk factors including hypertension and hyperlipidaemia not related to completion.

^b^ Higher VO_2_ max [55,62,73].

**Table 7:** Factors from other categories showing no evidence of relationship with uptake / completion of CR programmes [references]

**Category**	**Factors**
**Language barriers**[76]	Completion only
**Behaviours: personal choices & cultural preferences**[60,71]	*Uptake* – past smoker
	*Completion* – calorie consumption
**Physical wellbeing**[31,34,35,37,38,40,43,47,50,52][55,60,67-69,71,74-76,78]	*Uptake* - Number of cardiac events; previous history of stroke/TIA; unstable angina; congestive heart failure; total cholesterol; triglycerides; ECG ST depression or elevation; creatine kinase levels; tropinin levels; number of risk factors present; medication for angina / arrhythmia / calcium antagonists; diuretics; level of co-morbidity
	*Completion* - Previous history of MI / CABG / PCI / PTMC / valvoplasty; MI; PCI procedure; dyslipidemia; ejection fraction; fasting blood glucose; heart rate; high density and low density lipoprotein cholesterol; hypertension; hyperlipidemia; total cholesterol; triglycerides; treadmill velocity; functional capacity; heart rate after exercise; quality of life’ physical health score (SF36); waist circumference
**Balancing & integrating health care needs with everyday life**[32,56]	*Uptake* - Losses in life; time stress
**Culture**[67,70]	Country of birth; ethnicity
**Attitudes to rehabilitation**[48]	Greater concerns about harmful effects of exercising
Social support [63]	*Uptake* – emotional support
**Formal support**[43]	*Uptake* - Length of hospital stay

As with the key themes, concordance between the factors that informed the qualitative framework [17] and those reported in the quantitative data was in general poor. Patient reported factors in for example ‘personal choices and cultural preferences’ related to unwillingness to change habits and feelings of resentment from enforced changes in lifestyle. These were not addressed by quantitative studies, which only referred to smoking status.

## Discussion

To our knowledge this is the first systematic review of the cardiovascular literature that attempts to map quantitatively defined factors to those that patients perceive to be important in deciding upon lifestyle behaviour change. The review is enhanced by this approach as it demonstrates the divergent foci of the two methodologies and reinforces the need to examine both bodies of evidence.

The value of our review is that it can inform a consideration of the skills required to fulfil increasingly active public health agendas across developed countries. For example, the UK Department of Health’s ‘Every Contact Counts’ (http://www.dh.gov.uk/health/2012/01/forum-response/initiative accessed 13/1/2012) is one of a number of policy led changes to the delivery of health promotion and prevention [79,80]. A key requirement is for front line health care providers to seize every opportunity to promote and support individuals to adopt healthy lifestyles. Understanding key factors that influence lifestyles may improve the quality of these interactions and so increase initiation of lifestyle change.

### Comparison with existing literature

An earlier review of factors associated with uptake of cardiac rehabilitation [19] found age, deprivation, beliefs and physician recommendations to be the main predictors. However in our review the majority of studies found no relationship between age and uptake of cardiac rehabilitation and we would suggest other factors such as referral patterns (potentially favouring younger patients) and lack of work commitments (in older patients) might contribute to this unclear picture. As in the Cooper review [19] we also found strong evidence that beliefs have an important role in influencing patient decisions about lifestyle change, as was support from family and friends. Previous reviews have been limited to uptake (often termed ‘attendance’) at cardiac rehabilitation. We have extended this to include completion thus enabling us to consider the trajectory of influences on lifestyle change beyond the initial stages. Although we could not glean much from the programme completion data, it does appear that in certain circumstances (depression, personal beliefs and possibly partner status) factors that deter uptake may also increase the likelihood of programme drop-out.

Ideally intervention studies aiming to support lifestyle behaviour change should attempt to address a range of potential barriers. A brief examination of previous intervention studies that have specifically sought to address barriers to lifestyle change reveals limited awareness of the main predictors of uptake of lifestyle change. Assessment of social (family) support and education were reported in two studies [14,81]. Mood problems, transport, costs, and beliefs lacked mention despite them having a key role in influencing uptake and completion of lifestyle change.

### Summary of main findings

Our initial hypothesis that key patient perceived barriers (relating to emotions, psychological beliefs, information / communication needs, support from family and friends, and transport and other costs) would be predictive of uptake of lifestyle behaviour change appears to hold true. In particular, problems with transport and attribution of greater consequences and more symptoms to illness were most consistently predictive of uptake. Other factors provide less clear evidence as to their role in either uptake or completion despite, as is the case with factors related to physical wellbeing, extensive examination. In comparison with uptake, completion of lifestyle behaviour change has received relatively little attention. From the data that is available, there is a suggestion that some factors mainly predictive of uptake are not necessarily the same as those predicting completion.

Although representation of patient reported factors in the quantitative studies was in general not good, in some instances this may not be problematic. For example, the relationship between depression and, to a lesser extent anxiety, and uptake / completion was a strong feature of the cohort studies but not the qualitative literature. Rates of co-morbid depression in adults with diabetes and heart disease are thought to be double those seen in non-affected populations [82,83]. The increased risk of depression and thereafter poorer outcomes reinforces the importance of recognition of mood problems in these populations. The apparent lower profile of these types of mood problems in the qualitative literature may be due to self selection (of those with low mood out of interviewing) or stigma of personal disclosure during interviews.

Conversely, for other categories such as ‘formal support’, ‘friends and family support’, and ‘social support’, cohort studies had failed to address issues that appear to be of central importance to patients. Thus network size and marital status are reported but perceived support not.

### Strengths and limitations

Despite our aim to examine lifestyle change in programmes other than cardiac rehabilitation, this was not achieved. We used the same search strategy to identify qualitative studies examining patient perceived influences in uptake of lifestyle change and found studies relating to diabetes and hypertension. This perhaps highlights the paucity of research into long-term conditions and changing lifestyle behaviours through formal programmes. In spite of this limitation we consider that these findings will be generalisable to the primary care population with risk factors for cardiovascular disease for two reasons. Firstly, the categories were derived from studies representing a broad range of cardiovascular related conditions. Secondly studies included in the current review reported patients attending outpatient cardiac rehabilitation who would therefore experience similar practical and social difficulties as those attempting to attend lifestyle change programmes in the community. Although the studies were of adequate quality, many failed to define what they meant by attendance and participation. We therefore assumed that the presence of a non-attending comparator group meant that these studies related to uptake. However, we do not know if uptake related to attendance at one or multiple sessions and we would question the meaningfulness of including data where attendance at for example one session was commonplace.

We did not formally investigate the possibility of publication bias because the majority of studies reported multiple factors. However, we did observe a broad range of significant and non-significant findings indicating little evidence for publication bias.

The studies reported in the current review that were derived from the systematic review by Cooper et al. [19] were not assessed for quality by either ourselves or in the previous review. We intended for our work to be an update and expansion of the original review and therefore did not re-examine earlier studies. This may have affected the interpretation of the results in some categories. For example, the ‘information and communication’ the majority of studies indicating that lower educational status correlated with poor uptake were reported in the Cooper review [19] but later studies have not confirmed this relationship.

## Conclusions

In conducting this review, our aim was to gather robust information that would support the development of a lifestyle assessment to be used at the point of for example referral to lifestyle support. We consider that the evidence gathered supports the findings from our previous qualitative review that at least five areas (emotional status, access to transport and cost of programmes, knowledge, understanding and beliefs about the condition and healthy lifestyles, and the quality of personal support) are influential in lifestyle change. Narrowing down the field of factors that influence lifestyle behaviour change should support health care providers to garner the knowledge and skills required to achieve more productive consultations. Further work is required to formalise these areas into an approach that can characterise patient responses in these areas and guide decision making about the most suitable type (formal group programme versus individual support) and level (supported change versus self-managed change) of support. Further research is needed to examine the nature of potential relationships between the factors identified in this and our previous review, and completion of lifestyle change programmes other than cardiovascular rehabilitation. In the meantime we recommend that health care providers use the information provided in this review (see summary in Table 8) to guide decisions about referral to lifestyle support.

**Table 8:** Summary of clinical messages from key themes

**Themes**	**Main message**
**Emotions** Depression, anxiety, stress, poor motivation, lack of confidence, embarrassment.	Patients with depression are less likely to take up both the offer of lifestyle change support and complete any programmes. Depression is linked with obesity and poor health outcomes. Other more subtle emotional barriers may also deter patients from changing lifestyles.
**Psychological & spiritual beliefs** Beliefs about role of health behaviours and extent of physical recovery	Patients who do not consider that lifestyles influence health or that they can manage their risks are less likely to take up the offer of lifestyle change support. Providing patients with evidence on how lifestyles reduce risks may encourage patients to re-think their beliefs.
**Information & communication** Lack of knowledge and misperceptions about purpose of healthy lifestyles, poor understanding of communication about risks and diet	Lack of knowledge about the role of lifestyles in managing cardiovascular risk may deter patients from taking up lifestyle support. Poor knowledge may engender misperceptions and so improving knowledge may challenge beliefs.
**Friends & family support** Close social support appears to impact on attempts to change and maintain healthy lifestyles.	Good support from family and friends can facilitate uptake of lifestyle behaviour change. Encouraging partners to attend Health Checks and annual reviews may increase uptake of lifestyle change programmes.
**Transport & cost** Difficulties with access to specific centres to undertake rehabilitation. Costs associated with transport and daily costs of living	Transport and cost issues are a significant barrier for patients. Referral to lifestyle support should be coupled with questioning about accessibility and affordability. Referral to social services may help.

## Competing interests

The authors declare that they have no competing interest.

## Authors' contributions

JM wrote the study protocol, led the review process and writing of the manuscript. CC was involved in all stages of the data extraction and analysis and assisting in writing the manuscript. KH assisted in preparing the study protocol and was involved in the data extraction, analysis and writing of the manuscript. SH was involved in data extraction and analysis and contributed to the content of the manuscript. AH conceived the idea for the review, contributed to writing the protocol and contributed to the manuscript preparation. All authors have read and approved the final manuscript.

## Authors' information

Jenni Murray, BSc, MSc, Phd, Senior Research Fellow.

Cheryl Craigs, PgDip, BSc, MSc, Doctoral Student.

Kate Hill, BSc, MSc, Phd, Senior Research Fellow.

Stephanie Honey, BSc, MSc, Phd, Research Fellow.

Allan House BSc. MBBS, MRCP, MRCPsych DM, Professor of Liaison Psychiatry.

## Declarations

Ethical approvals: not required.

## Pre-publication history

The pre-publication history for this paper can be accessed here:

http://www.biomedcentral.com/1471-2261/12/120/prepub

## Supplementary Material

Additional file 1 **Appendix 1.** Quality assessment. Click here for file
