# Supplementary material for: A systematic review of patient reported factors associated with uptake and completion of cardiovascular lifestyle behaviour change
Source: BMC Cardiovasc Disord. 2012 Dec 8;12:120. doi: 10.1186/1471-2261-12-120 (PMC3522009; doi:10.1186/1471-2261-12-120)
Supplement: Additional file 1 — Appendix 1. Quality assessment. [file 1471-2261-12-120-S1.docx]

### Appendix 1: Quality assessment

|  | Item | No. studies* [References] | |
| --- | --- | --- | --- |
|  | Objective was clearly stated | 31 | [[46-57](#_ENREF_46), [59-60](#_ENREF_59), [62-78](#_ENREF_62)] |
|  | Recruitment method was clear | 27 | [[46-49](#_ENREF_46), [51-53](#_ENREF_51), [57-76](#_ENREF_57), [78](#_ENREF_78)] |
|  | Participant inclusion/exclusion criteria were clearly described | 24 | [[48-57](#_ENREF_48), [60-61](#_ENREF_60), [63-64](#_ENREF_63), [66-67](#_ENREF_66), [69-72](#_ENREF_69), [74-76](#_ENREF_74), [78](#_ENREF_78)] |
|  | Cardiac rehabilitation/advice was clearly described | 20 | [[46-47](#_ENREF_46), [49-50](#_ENREF_49), [52](#_ENREF_52), [54](#_ENREF_54), [56](#_ENREF_56), [59](#_ENREF_59), [62](#_ENREF_62), [65-71](#_ENREF_65), [73-74](#_ENREF_73), [76](#_ENREF_76), [78](#_ENREF_78)] |
|  | Period of follow-up was clear** | 23 | [[46-47](#_ENREF_46), [50-52](#_ENREF_50), [54-57](#_ENREF_54), [59-60](#_ENREF_59), [63-66](#_ENREF_63), [69-71](#_ENREF_69), [73](#_ENREF_73), [75-78](#_ENREF_75)] |
|  | Procedure for obtaining data was clearly described | 28 | [[47-48](#_ENREF_47), [50-60](#_ENREF_50), [62-71](#_ENREF_62), [75-78](#_ENREF_75)] |
|  | Outcomes were clearly defined | 25 | [[46-57](#_ENREF_46), [62-69](#_ENREF_62), [71-72](#_ENREF_71), [76-78](#_ENREF_76)] |
|  | Methods for measuring outcomes and factors were clearly described | 26 | [[46-57](#_ENREF_46), [59](#_ENREF_59), [63-64](#_ENREF_63), [66-72](#_ENREF_66), [74](#_ENREF_74), [76-78](#_ENREF_76)] |
|  | Power calculation was included | 2 | [[54](#_ENREF_54), [68](#_ENREF_68)] |
|  | Statistical methods were described | 28 | [[47-49](#_ENREF_47), [51-57](#_ENREF_51), [60-72](#_ENREF_60), [74-78](#_ENREF_74)] |
|  | Statistical methods were appropriate | 28 | [[46-55](#_ENREF_46), [59-65](#_ENREF_59), [68-78](#_ENREF_68)] |
|  | Percentage successful recruitment, loss to follow-up or missing data were reported** | 24 | [[46](#_ENREF_46), [51-52](#_ENREF_51), [54-61](#_ENREF_54), [63-64](#_ENREF_63), [66-68](#_ENREF_66), [71-78](#_ENREF_71)] |
|  | Sufficient baseline data by participant group were included | 31 | [[46-71](#_ENREF_46), [74-78](#_ENREF_74)] |
|  | Sufficient results were reported to assess conclusions | 33 | [[46-78](#_ENREF_46)] |

*Number of studies fully meeting each criterion

** A number of studies were not applicable for this criterion
